# A case of multiple median sternotomy for infection and expanding hematoma in 10 years

**DOI:** 10.1186/s43044-023-00411-z

**Published:** 2023-10-06

**Authors:** Tomohiro Nakajima, Yutaka Iba, Tsuyoshi Shibata, Nobuyoshi Kawaharada

**Affiliations:** Department of Cardiovascular Surgery, Sapporo Medical University School of Medicine, South-1, West-16, Chuo-ku, Sapporo 060-8543 Japan

**Keywords:** Infective endocarditis, Expanding hematoma, Repeated median sternotomy

## Abstract

**Background:**

After a median sternotomy, mediastinitis may develop, necessitating reopening of the chest. Rarely, reoperation due to hematoma after cardiovascular surgery is experienced. In the present case, we experienced a patient who initially had mediastinitis, but later developed a chronic hematoma and underwent multiple surgeries.

**Case presentation:**

The patient was a 40-year-old man who underwent aortic valve replacement for a bicuspid aortic valve and a graft for a dilated ascending aorta. Postoperatively, he developed hematoma in the anterior mediastinum on multiple occasions with repeated episodes of infection that required multiple median sternotomies.

**Conclusions:**

We reported our experience with a rare case of multiple median sternotomies. In the early stage, mediastinitis due to infection was observed, and in the late stage, mediastinal dilatation due to hemorrhage was observed.

## Background

The patient was a 40-year-old man who underwent aortic valve replacement for a bicuspid aortic valve and a graft for a dilated ascending aorta. Postoperatively, he developed hematoma in the anterior mediastinum on multiple occasions with repeated episodes of infection that required multiple median sternotomies. This rare case is reported in view of the difficulties encountered in diagnosis and treatment.

## Case presentation

We often encounter infection in an ascending aortic graft, which is difficult to treat. The reason is that an infection of the ascending aorta requires reoperation for artificial vessel replacement, aortic mesh filling, and long-term antibiotic therapy, which can be fatal if not effective. This report describes a case of mediastinitis one year after ascending aorta graft surgery. Postoperatively, the patient developed mediastinal hematoma on multiple occasions that was difficult to diagnose and entailed multiple median sternotomies. In this report, we summarize this rare case and the relevant literature.

The patient was a 40-year-old otherwise healthy man who was found to have a heart murmur on physical examination. Before the first surgery, he had NYHA2 heart failure symptoms due to aortic regurgitation. The diameter of the ascending aorta was 50mm. Echocardiography confirmed a bicuspid aortic valve with severe aortic regurgitation and dilatation of the ascending aorta, both of which were considered indications for surgery. He underwent aortic valve replacement with a 23-mm SJM Regent mechanical valve and repair of the ascending aorta using a 26-mm Gelweave graft at another hospital (operation 1). The patient was discharged from hospital without any problems and followed up as an outpatient.

**Fig. 1:**
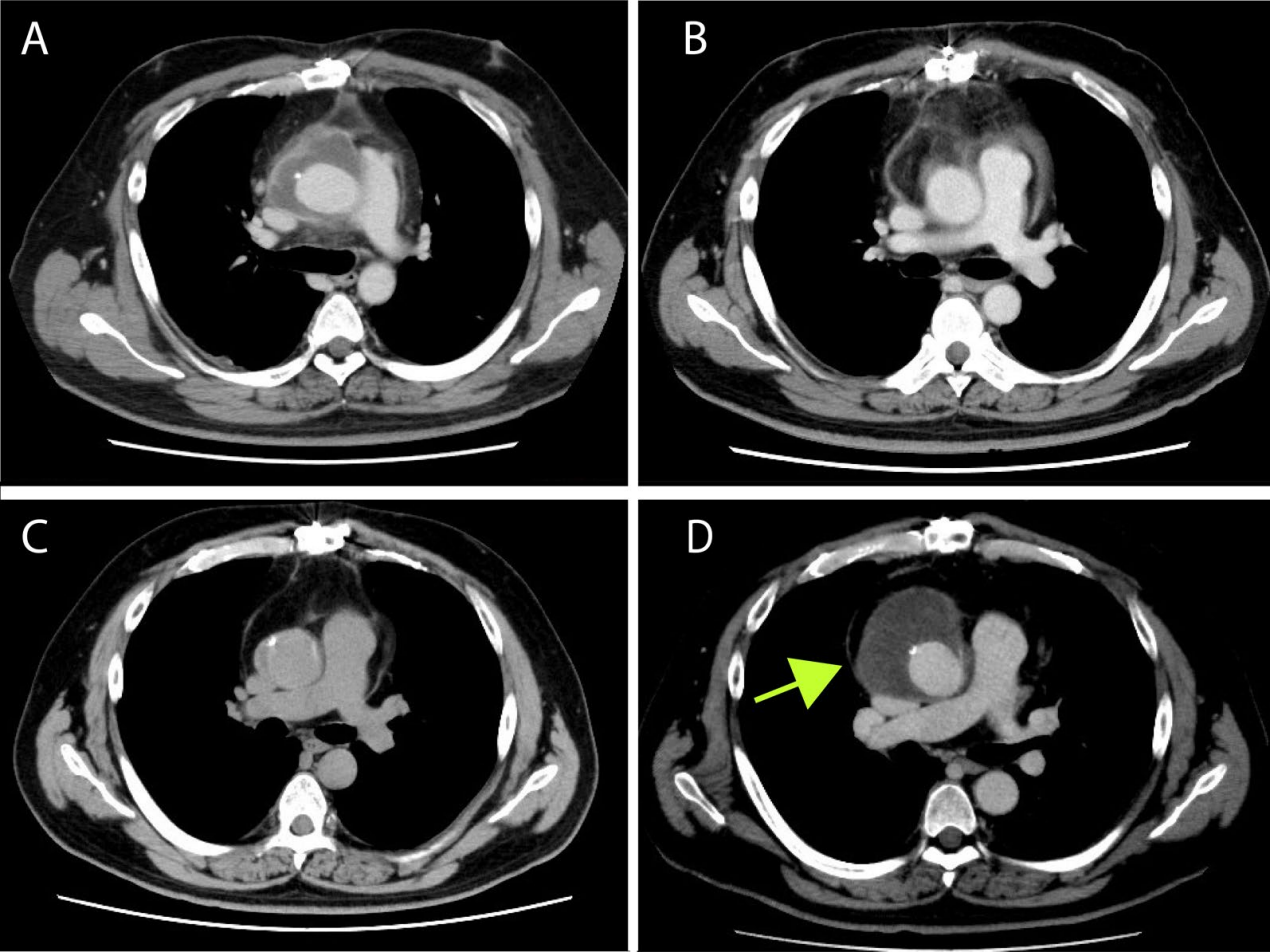
Findings on CT scans during the first 5 years after the initial surgery. **a** POY 1. Residual fluid was detected around the ascending aortic graft. An infected graft was diagnosed based on a positive blood culture and laboratory test results. **b** POY 1. A CT scan obtained after omentoplasty. **c** POY 3. A CT scan showing that the mediastinum was not filled. **d** POY 4 Residual fluid was seen in the anterior mediastinum around the ascending aortic graft (green arrow). CT, computed tomography; POY, postoperative year

One year later, he presented with a fever of 38°C, a white blood cell count of 10,300/μL, and a C-reactive protein level of 18.3 mg/dL. CT showed mediastinitis (Fig. 1A). A blood culture revealed methicillin-resistant *Staphylococcus aureus* (MRSA). We performed a redo ascending aortic replacement and aortic valve replacement (operation 2). Prior to re-sternal median incision, the femoral arteriovenous vein was exposed. The next day, we confirmed hemostasis and performed omentopexy around the ascending graft (operation 3) (Fig. 1B). The patient was discharged from hospital without any problems. In POY 3, a computed tomography (CT) scan showed that the mediastinum was not filled (Fig. 1C). The mediastinum was found to be filled with greater omentum, but there was still space for fluid to accumulate there. A further CT scan obtained in POY 4 showed fluid retention around the ascending aortic graft (Fig. 1D), which was considered to be a seroma. The surrounding tissue was removed (operation 4).

**Fig. 2:**
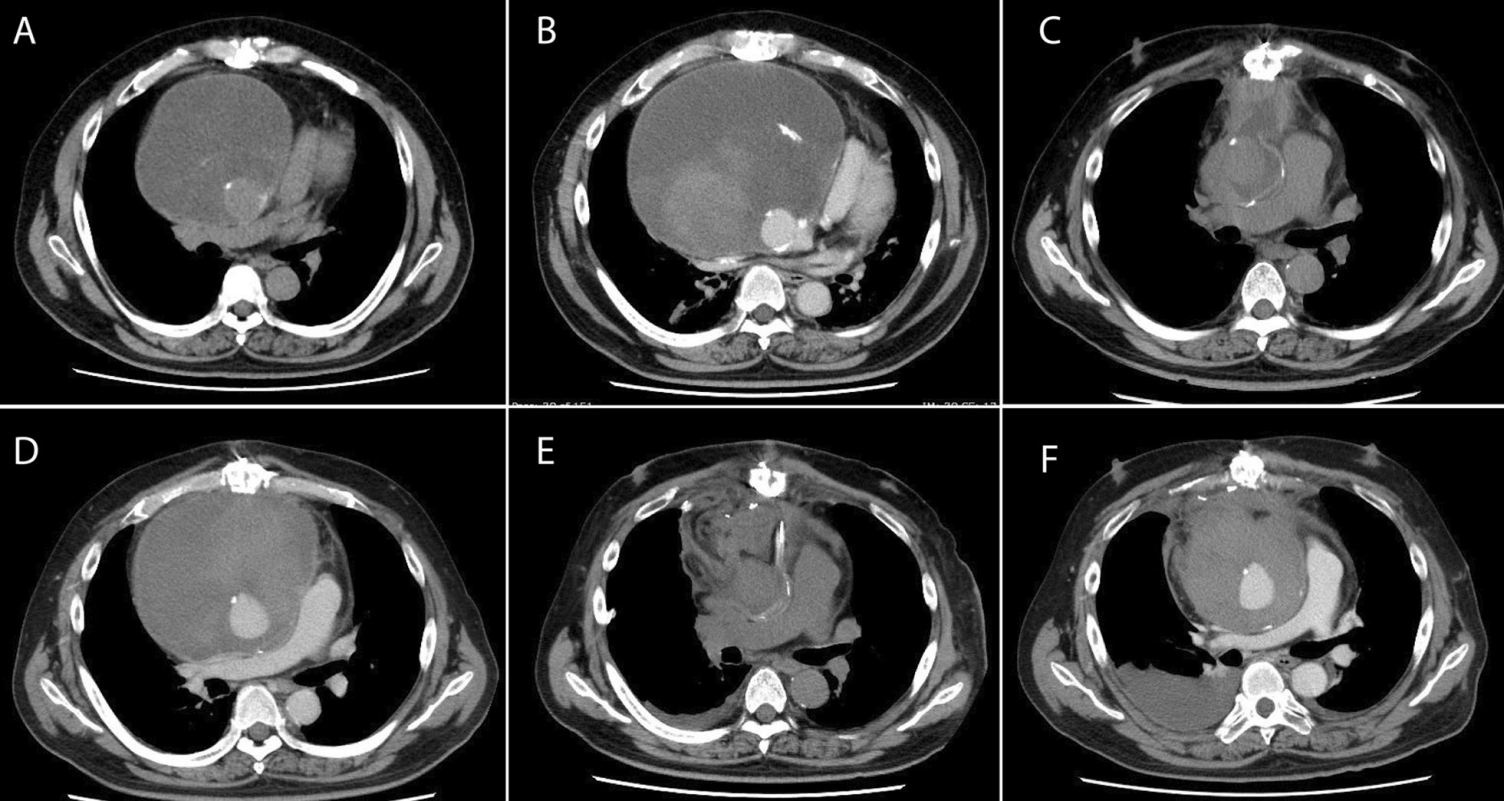
Findings on CT scans from years 6 to 9 after the initial surgery. **a** POY 6. A mass was detected around the ascending aorta. **b** POY 7. A 100-mm mass was detected around the ascending aorta and the ascending aortic graft was replaced. **c** A CT scan obtained immediately thereafter showed slight effusion around the ascending aorta. **d** Two months later, fluid retention was again observed around the ascending aorta. **e** The hematoma capsule was removed, and after confirming hemostasis, the left vastus lateralis muscle was filled into the anterior mediastinum. **f** Six months later, further hematoma was found in the mediastinum. CT, computed tomography; POY, postoperative year

The patient was ambulatory and did well thereafter. A CT scan obtained in POY 6 (Fig. 2A) showed fluid in the tissue around the graft but no signs of infection, and follow-up was continued. In POY 7, more fluid was found in the tissue around the graft (Fig. 2B), and fluid around the ascending aorta was compressing the heart. The left atrium was compressed, resulting in poor pulmonary venous return and decreased oxygenation. Airway compression was expected during induction of general anesthesia. After induction of general anesthesia, ECMO was immediately administered, and then, the chest was opened to remove the hematoma around the ascending aorta, which stabilized general oxygenation and hemodynamic status. Oozing was noted in the operation, indicating that the graft had failed; the graft was replaced with a 26-mm Triplex, and the patient was stable thereafter (Fig. 2C) (operation 5). The oozing ran the entire length of the artificial graft from the black line. Since the oozing was from the entire length of the artificial vessel, it was impossible to repair and had to be replaced. Two months later, fluid retention was again observed around the ascending aorta (Fig. 2D), and a diagnosis of expanding hematoma was made. After a further week, the hematoma capsule was removed (operation 7), and hemostasis was confirmed. On the following day, the left vastus lateralis muscle was filled into the anterior mediastinum (operation 8) (Fig. 2E) in collaboration with the plastic surgeons, after which the patient was discharged. However, 6 months later, he was found to have another hematoma in the mediastinum (Fig. 2F), which was removed (operation 9).

**Fig. 3:**
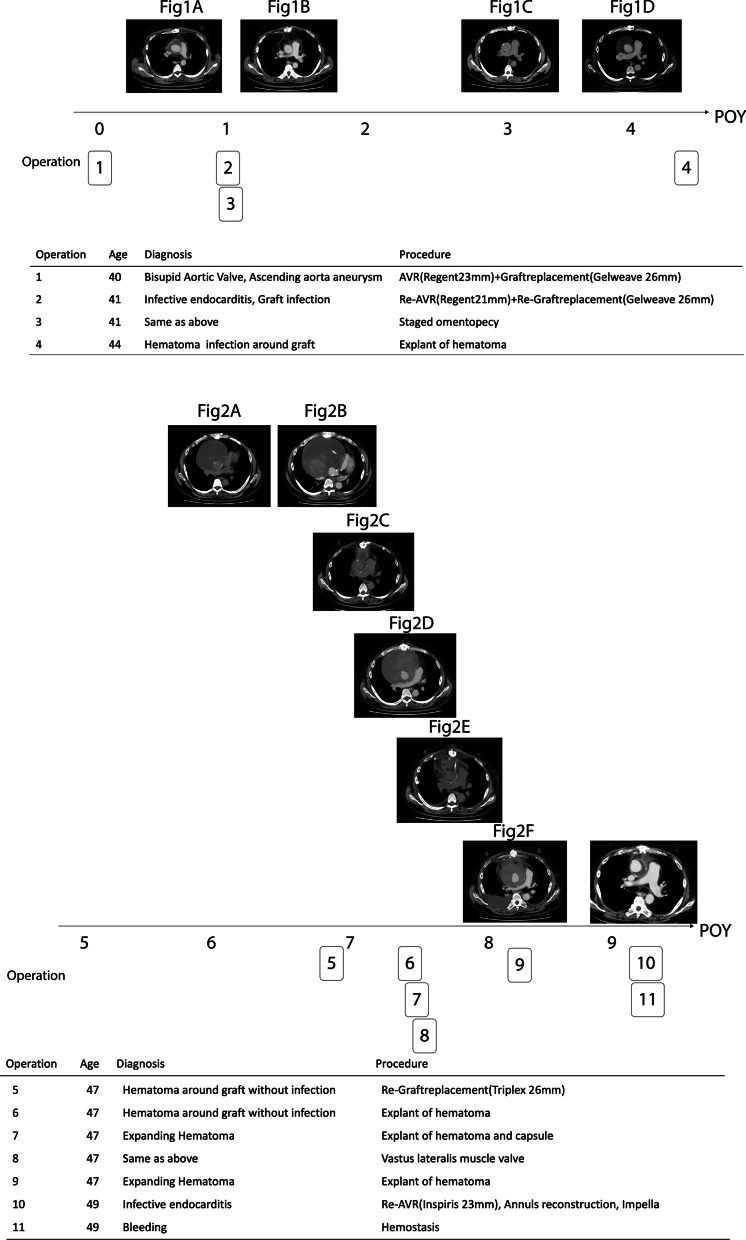
Disease course, findings on computed tomography, diagnoses, and operations performed during years 1–9 after the initial surgery

One year later, he was admitted to hospital with sepsis caused by MRSA. He had a fever of 38 degrees Celsius three days prior to admission. The seating of the aortic valve was observed to be unstable (operation 10). Therefore, the aortic valve was urgently replaced with a 23-mm Inspiris valve and a diagnosis of infective endocarditis was made (operation 11). The infection could not be controlled, and the patient died on postoperative day 20. The family did not wish to have a postmortem study. The patient’s disease course and details of the operations performed are shown in Fig. 3.

## Discussion

Cases requiring surgery for bicuspid aortic valve and ascending aorta aneurysm are not particularly rare [1]. We occasionally encounter patients in whom mediastinitis develops after open heart surgery and placement of a thoracic aortic prosthesis, which can be treated by omentoplasty to the pleural cavity [2]. After that, the patient experienced recurrences of mediastinal hematoma and underwent a total of multiple sternotomies.

Figure 3 shows the details of the initial surgery and removal of a mediastinal hematoma 1 year later. The patient had been healthy from birth and was not immunodeficient. The portal of entry for MRSA was unknown. Until then, the postoperative course was uneventful (Fig. 1C). However, mediastinal hematoma was detected 4 years after the initial surgery. There were no signs of infection at this time, but a white turbid brittle solid area was observed around the ascending aortic graft, which was considered to be a sclerotic hematoma. In addition, the artificial blood vessel periphery and mediastinal tissue were not adhered and the artificial blood vessel itself was easily exposed. Given that the aortic valve had been replaced with a mechanical valve, oral warfarin was administered at a dosage that maintained a prothrombin time/international normalized ratio in the range of 2.0–3.0. Therefore, the direct cause of the hematoma was unknown. As shown in Fig. 3, there were no untoward problems until that time. However, the mediastinal hematoma was subsequently noted to be enlarging. Considering that the mediastinum is an enclosed space, we anticipated that ongoing bleeding would be unlikely and continued to observe the patient. However, one year later, the mediastinal hematoma was found to have expanded further and respiratory failure was recognized as a symptom by compressing the ventricle. The hematoma was resected. Furthermore, the ascending aortic graft was found to be disrupted and was replaced. Two months later, mediastinal hematoma was detected again and diagnosed to be expanding hematoma caused by longstanding mediastinal infection. The hematoma capsule was removed to fill the previous mediastinum for the purpose of infection control, and the left latissimus dorsi was taken off together with plastic surgery and filled in the anterior mediastinum by anastomosing the right internal thoracic artery [3]. However, 2 months later, the hematoma reappeared and was again removed. Infection was also detected at this time. Further surgical intervention became difficult at this point, so only hematoma removal was performed. Hematology results (CBC, Plt, and Fibrinogen) did not reveal any clotting problems. We maintained the patient’s prothrombin time/international normalized ratio at a slightly low value of about 1.5 and continued to follow him closely. One year later, we detected another mediastinal hematoma and an infected valve prosthesis. Artificial valve replacement was performed, but the patient subsequently developed septic shock and died. No adhesions were found around the ascending aorta graft area at any time in this patient. It is possible that certain congenital or acquired host factors or an acquired coagulation disorder [4] caused the problems encountered in this case, but this could not be confirmed.

## Conclusions

We reported our experience with a rare case of multiple median sternotomies. In the early stage, mediastinitis due to infection was observed, and in the late stage, mediastinal dilatation due to hemorrhage was observed.

## Data Availability

Not applicable.
